# Choosing the right reporting guideline in veterinary research: an editorial for authors and editors

**DOI:** 10.4142/jvs.26903

**Published:** 2026-09-18

**Authors:** Pan Dong Ryu

**Affiliations:** College of Veterinary Medicine, Seoul National University, Seoul 08826, Korea.

## Abstract

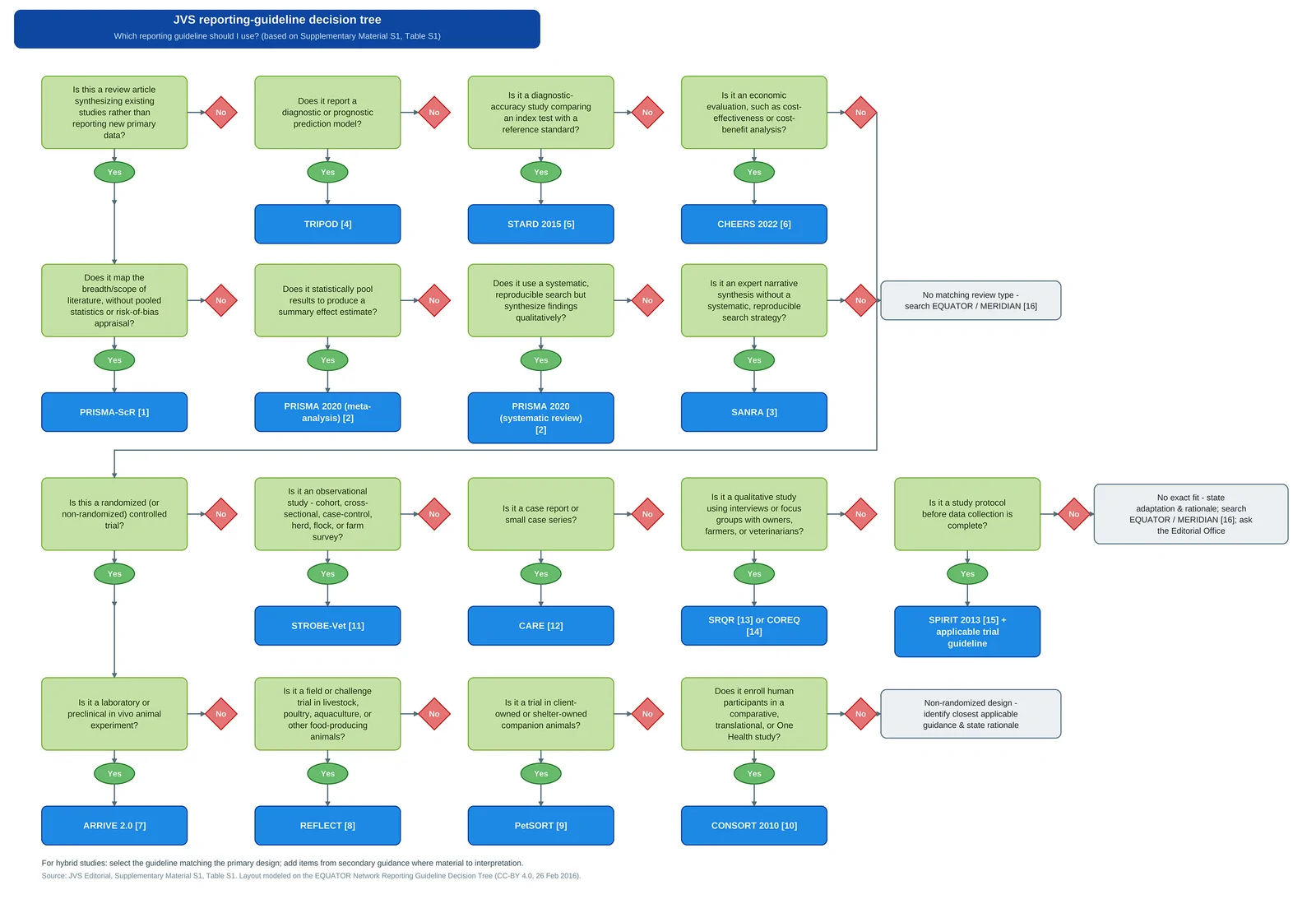

## REPORTING COMPLETENESS IS A SCIENTIFIC ISSUE

The reporting guidelines define the minimum information readers need to understand, critically evaluate, and reproduce health research [1]. They are often treated as a final submission requirement, but framing is too narrow. A checklist can expose the design choices that are otherwise easily lost during drafting: the unit of allocation, the handling of clustered observations, the basis for sample-size decisions, the conditions of animal housing, and the pathway through which participants or owners entered a study.

For veterinary science, incomplete reporting has amplified the consequences. Studies can span species, production systems, client-owned animals, laboratory settings, and One Health contexts. The unit that matters analytically may be an animal, cage, litter, pen, herd, flock, farm, or owner. A report that does not specify this unit can misstate the precision and impair risk-of-bias assessment. Therefore, the question is not whether reporting guidance adds administrative work; it is whether the research record preserves the information needed for its claims to remain useful.

## THE VETERINARY GAP IS NOT SOLVED USING A GENERIC CHECKLIST

Human-medicine standards provide an indispensable foundation but directly transferring them can miss the features that shape veterinary inference: clustering, husbandry, client-mediated consent, and welfare decisions such as euthanasia. A family of veterinary-specific extensions now addresses these realities. ARRIVE 2.0 (Animal Research: Reporting of *In Vivo* Experiments) governs laboratory and preclinical animal research [2]. REFLECT (REporting guidelines For randomized controLled trials for livEstoCk and food safety) covers livestock and food-safety trials [3]. PetSORT (Pet Standards Of Reporting Trials) applies to client- and shelter-owned companion-animal trials and makes euthanasia an explicit reporting item [4]. STROBE-Vet, the veterinary extension of STROBE (STrengthening the Reporting of Observational Biological studies in Epidemiology), is the default for observational designs [5]. STARD (STAndards for Reporting Diagnostic accuracy studies) covers diagnostic-accuracy studies [6], and CARE (CAse REport) covers case reports [7]. These differences are methodological, not cosmetic; specifying whether allocation and analysis occurred at the animal, pen, or herd level is necessary to judge clustering and the validity of the estimated uncertainty.

The same problem arises with evidence syntheses. A narrative review, a scoping review, a systematic review, and a meta-analysis are not interchangeable labels. SANRA (a ScAle for the quality assessment of Narrative Review Articles) governs narrative reviews [8], PRISMA-ScR (Preferred Reporting Items for Systematic Reviews and Meta-Analyses extension for Scoping Reviews) governs scoping reviews [9], and PRISMA 2020 governs systematic reviews and meta-analyses [10]. Mislabeling a narrative overview as a systematic review creates expectations of reproducible searching, eligibility criteria, and appraisal that the work may not meet.

## A PRACTICAL PATHWAY: CHOOSE EARLY, CHOOSE THE PRIMARY DESIGN

Table 1 summarizes a decision pathway synthesized from the *JVS* (*Journal of Veterinary Science*) Instructions to Authors and the guideline documents cited here; it is an editorial heuristic, not a validated instrument. Authors should first identify whether the manuscript synthesizes the existing literature or reports primary data, and for reviews, distinguish a narrative overview, scoping review, systematic review, and meta-analysis according to the aims and methods. For primary research, the central design determines the guideline: laboratory or preclinical trials use ARRIVE 2.0; livestock and food-animal trials use REFLECT; companion-animal trials use PetSORT; observational studies default to STROBE-Vet; diagnostic-accuracy studies use STARD; and case reports use CARE. A full stepwise algorithm covering additional designs, including prediction models, economic evaluations, qualitative research, protocols, and hybrid studies, is provided in Supplementary Data 1 and Supplementary Table 1.

**Table 1:** Reporting-guideline crosswalk for common veterinary manuscript designs

Manuscript design	Veterinary-specific or preferred guideline
Laboratory/preclinical *in vivo* animal research	ARRIVE 2.0 [2]
Livestock or food-animal randomized trial	REFLECT [3]
Client-/shelter-owned companion-animal clinical trial	PetSORT [4]
Observational veterinary study	STROBE-Vet [5]
Diagnostic accuracy study	STARD [6]
Case report or case series	CARE [7]
Narrative review	SANRA [8]
Systematic review/meta-analysis	PRISMA 2020 [10]
Scoping review	PRISMA-ScR [9]

ARRIVE, Animal Research: Reporting of *In Vivo* Experiments; REFLECT, REporting guidelines For randomized controLled trials for livEstoCk and food safety; PetSORT, Pet Standards Of Reporting Trials; STROBE-Vet, STrengthening the Reporting of OBservational studies in Epidemiology – Veterinary; STARD, STAndards for Reporting Diagnostic accuracy studies; CARE, CAse REport; SANRA, a ScAle for the quality assessment of Narrative Review Articles; PRISMA, Preferred Reporting Items for Systematic reviews and Meta-Analyses; PRISMA-ScR, Preferred Reporting Items for Systematic reviews and Meta-Analyses extension for Scoping Reviews.

Hybrid studies should declare a primary design and use the matching guidelines as the reporting backbone, adding items from secondary guidance where they materially affect validity. A companion-animal trial with a diagnostic-accuracy component, for example, should use PetSORT for trial reporting and incorporate the relevant STARD items. When no checklist is an exact fit, authors should state this openly in the Methods and identify the closest available standard; Supplementary Data 1 and Supplementary Table 1 also point to the EQUATOR (Enhancing the QUAlity and Transparency Of health Research) and MERIDIAN (Menagerie of Reporting guidelines Involving Animals) Networks for less common designs.

## DISCUSSION

Veterinary research requires reporting guidance that is both rigorous and navigable. The growth of veterinary-specific extensions signals methodological maturity, but it also imposes a selection burden for authors. A simple principle can reduce that burden: choose the guideline that best matches the primary design and population, then add secondary items that materially affect the interpretation. This principle respects the common foundation shared with human clinical and epidemiological reporting while preserving the features that make veterinary evidence distinctive.

No pathway can eliminate judgment. Novel, mixed, or emerging designs will sometimes fall between established checklists. In those cases, a transparent explanation is preferable to false precision. Authors should identify the closest guideline, explain the adaptations, and seek editorial advice early when uncertainty remains.

The intended outcome is not a longer administrative checklist. It is a clearer, more durable research record: one that lets readers understand what was done, reviewers assess the validity efficiently, and future investigators include veterinary evidence in synthesis and policy. Therefore, selecting the correct reporting guideline is a small early decision with a disproportionate return for veterinary science.

## SUPPLEMENTARY MATERIALS

Supplementary Data 1 Algorithm for selecting a reporting guideline for veterinary manuscripts

Supplementary Table 1 Stepwise selection of the reporting guidance for veterinary manuscripts

## References

[1] Simera I, Moher D, Hoey J, Schulz KF, Altman DG (2010). A catalogue of reporting guidelines for health research. Eur J Clin Invest.

[2] Percie du Sert N, Hurst V, Ahluwalia A, Alam S, Avey MT, Baker M (2020). The ARRIVE guidelines 2.0: updated guidelines for reporting animal research. PLoS Biol.

[3] O’Connor AM, Sargeant JM, Gardner IA, Dickson JS, Torrence ME, Dewey CE (2010). The REFLECT statement: methods and processes of creating reporting guidelines for randomized controlled trials for livestock and food safety. J Vet Intern Med.

[4] Ruple A, Sargeant JM, Selmic LE, O’Connor AM (2023). The standards of reporting randomized trials in pets (PetSORT): methods and development processes. Front Vet Sci.

[5] Sargeant JM, O’Connor AM, Dohoo IR, Erb HN, Cevallos M, Egger M (2016). Methods and processes of developing the STrengthening the Reporting of OBservational studies in Epidemiology-Veterinary (STROBE-Vet) statement. J Food Prot.

[6] Bossuyt PM, Reitsma JB, Bruns DE, Gatsonis CA, Glasziou PP, Irwig L (2015). STARD 2015: an updated list of essential items for reporting diagnostic accuracy studies. BMJ.

[7] Gagnier JJ, Kienle G, Altman DG, Moher D, Sox H, Riley D (2014). The CARE guidelines: consensus-based clinical case report guideline development. J Clin Epidemiol.

[8] Baethge C, Goldbeck-Wood S, Mertens S (2019). SANRA-a scale for the quality assessment of narrative review articles. Res Integr Peer Rev.

[9] Tricco AC, Lillie E, Zarin W, O’Brien KK, Colquhoun H, Levac D (2018). PRISMA extension for Scoping Reviews (PRISMA-ScR): checklist and explanation. Ann Intern Med.

[10] Page MJ, McKenzie JE, Bossuyt PM, Boutron I, Hoffmann TC, Mulrow CD (2021). The PRISMA 2020 statement: an updated guideline for reporting systematic reviews. BMJ.

