# Supplementary material for: Choosing the right reporting guideline in veterinary research: an editorial for authors and editors
Source: J Vet Sci. 2026 Sep 18;27(5):e76. doi: 10.4142/jvs.26903 (PMC13655553; doi:10.4142/jvs.26903)
Supplement: Supplementary file 1 [file jvs-27-e76-s001.doc]

**Supplementary Data 1.** Algorithm for selecting a reporting guideline for veterinary manuscripts

First identify the primary design and objective of the manuscript. Follow the branch that matches that primary design. For hybrid studies, use the primary-design guideline as the reporting backbone and add items from secondary guidance when they materially affect validity or interpretation.

**Quick-reference decision flow**

**Decision point:** Review article without new primary data

**Action:** Identify the review type: scoping review → PRISMA-ScR (Preferred Reporting Items for Systematic reviews and Meta-Analyses extension for Scoping Reviews) [S1]; meta-analysis → PRISMA 2020 [S2]; systematic review without pooling → PRISMA 2020 [S2]; narrative review → SANRA (a ScAle for the quality assessment of Narrative Review Articles) [S3].

**Decision point:** Prediction model

**Action:** TRIPOD (Transparent Reporting of a multivariable prediction model for Individual Prognosis Or Diagnosis) [S4].

**Decision point:** Diagnostic test accuracy study

**Action:** STARD 2015 (STAndards for Reporting Diagnostic accuracy studies) [S5].

**Decision point:** Economic evaluation

**Action:** CHEERS 2022 (Consolidated Health Economic Evaluation Reporting Standards) [S6].

**Decision point:** Controlled trial

**Action:** Identify the study population: laboratory/preclinical animals → ARRIVE 2.0 (Animal Research: Reporting of *In Vivo* Experiments) [S7]; livestock, poultry, aquaculture, or food-producing animals → REFLECT (REporting guidelines For randomized controLled trials for livEstoCk and food safety) [S8]; client- or shelter-owned companion animals → PetSORT (Pet Standards Of Reporting Trials) [S9]; human participants in a comparative or One Health study → CONSORT 2010 (Consolidated Standards of Reporting Trials) [S10].

**Decision point:** Observational study

**Action:** STROBE-Vet (the veterinary extension of STROBE) [S11].

**Decision point:** Case report or case series

**Action:** CARE (CAse REport) [S12].

**Decision point:** Qualitative study

**Action:** SRQR (Standards for Reporting Qualitative Research) [S13] or COREQ (COnsolidated criteria for REporting Qualitative research) [S14].

**Decision point:** Study protocol

**Action:** SPIRIT 2013 (Standard Protocol Items: Recommendations for Interventional Trials) [S15], together with the applicable trial guideline.

**Decision point:** No exact fit/novel or hybrid design

**Action:** Search the EQUATOR (Enhancing the QUAlity and Transparency Of health Research) and MERIDIAN (Menagerie of Reporting guidelines Involving Animals) [S16] resources, use the closest applicable checklist, and explain adaptations or omissions in Methods; seek editorial advice if needed.
